# Allele Copy Number and Underlying Pathology Are Associated with Subclinical Severity in Equine Type 1 Polysaccharide Storage Myopathy (PSSM1)

**DOI:** 10.1371/journal.pone.0042317

**Published:** 2012-07-31

**Authors:** Rosie J. Naylor, Leanda Livesey, John Schumacher, Nicole Henke, Claire Massey, Kenny V. Brock, Marta Fernandez-Fuente, Richard J. Piercy

**Affiliations:** 1 Comparative Neuromuscular Diseases Laboratory, The Royal Veterinary College, London, United Kingdom; 2 Auburn University, Auburn, Alabama, United States of America; Brigham and Women's Hospital, Harvard Medical School, United States of America

## Abstract

Equine type 1 polysaccharide storage myopathy (PSSM1), a common glycogenosis associated with an R309H founder mutation in the glycogen synthase 1 gene *(GYS1)*, shares pathological features with several human myopathies. In common with related human disorders, the pathogenesis remains unclear in particular, the marked phenotypic variability between affected animals. Given that affected animals accumulate glycogen and alpha-crystalline polysaccharide within their muscles, it is possible that physical disruption associated with the presence of this material could exacerbate the phenotype. The aim of this study was to compare the histopathological changes in horses with PSSM1, and specifically, to investigate the hypothesis that the severity of underlying pathology, (e.g. vacuolation and inclusion formation) would (1) be higher in homozygotes than heterozygotes and (2) correlate with clinical severity. Resting and post-exercise plasma creatine kinase (CK) and aspartate aminotransferase (AST) enzyme activity measurements and muscle pathology were assessed in matched cohorts of PSSM1 homozygotes, heterozygotes or control horses. Median (interquartile range (IR)) resting CK activities were 364 (332–764) U/L for homozygotes, 301 (222–377) U/L for heterozygotes and 260 (216–320) U/L for controls, and mean (+/− SD) AST activity for homozygotes were 502 (+/116) U/L, for heterozygotes, 357 (+/−92) U/L and for controls, 311 (+/−64) U/L and were significantly different between groups (P = 0.04 and P = 0.01 respectively). Resting plasma AST activity was significantly associated with the severity of subsarcolemmal vacuolation (rho = 0.816; P = 0.01) and cytoplasmic inclusions (rho = 0.766; P = 0.01). There were fewer type 2× and more type 2a muscle fibres in PSSM1-affected horses. Our results indicate that PSSM1 has incomplete dominance. Furthermore, the association between plasma muscle enzyme activity and severity of underlying pathology suggests that physical disruption of myofibres may contribute to the myopathic phenotype. This work provides insight into PSSM1 pathogenesis and has implications for related human glycogenoses.

## Introduction

There are eleven glycogen storage myopathies recognized in humans, associated with the excessive accumulation of glycogen and/or amylopectin-like polyglucosan bodies within skeletal muscle fibres [1]. In addition, reports exist of other human cases with similar pathological features, where genetic causes are suspected, but unknown [2]. To date, the pathophysiology of many of these glycogenoses remains poorly understood. Patients may develop muscle cramps, weakness, exercise intolerance and rhabdomyolysis [3], but it remains unclear the degree to which these signs are associated with the physical disruption of muscle fibre ultrastructure by the accumulations of glycogen or polyglucosan [4], or with other disease mechanisms, such as diminished energy supply [5], [6], [7].

Type 1 equine polysaccharide storage myopathy (PSSM1) in horses is associated with a variety of clinical signs, including intermittent exertional rhabdomyolysis, muscle fasciculations, muscle atrophy, gait abnormalities and paresis [8]. PSSM1 was first described in detail by Valberg and colleagues in 1992 [9] and is associated with an autosomal dominant, missense, gain of function mutation (R309H) in the skeletal muscle glycogen synthase gene (*GYS1*) [10]. Suspected to be the result of an ancestral founder, the identical mutation is found in many different horse breeds worldwide, with some breeds, for example Percheron and Belgian Draft horses, having a particularly high disease prevalence [10], [11], [12]. As with some of the known human glycogenoses [13], [14], [15], [16], affected horses accumulate excessive glycogen and abnormal alpha-crystalline polysaccharide inclusions within skeletal muscle fibres, often in sub-sarcolemmal vacuoles or cytoplasmic inclusions [17], [18]. Consequently, study of this equine disease may provide important information regarding the pathogenesis of related human disorders, perhaps prompting novel treatment or management strategies.

In PSSM1-affected horses, pathological changes are most prominent in muscles with a high proportion of glycolytic (type 2×) fibres [19]. In addition to the non-specific, chronic myopathic changes (internalised nuclei and increased fibre size variation), affected horses accumulate glycogen and develop subsarcolemmal vacuolation and granular cytoplasmic polysaccharide inclusions, as they age [8], [9], [20], [21]. Fibres containing polysaccharide inclusions are often detected in clusters at the perimysium and epimysium [20], [22] perhaps due to regional differences in muscle fibre metabolism and/or in nutrient provision and blood supply. Typically, the inclusions consist of non-lysosomal bound intracytoplasmic aggregates of β particles of glycogen and polyglucosan bodies composed of less highly branched glycogen, interspersed with protein aggregates containing myoglobin, desmin and ubiquitin [8], [23].

Enhancing our understanding of the pathophysiology of this equine disease may provide further insight into the role of glycogen storage and polysaccharide inclusions in human glycogenoses; furthermore the high disease prevalence in horses [12] may facilitate such investigations. It remains unclear whether muscle cell damage in affected horses occurs as a result of an underlying metabolic defect (with polysaccharide inclusions being a co-incidental disease marker), or whether (and as in a murine model of Pompe disease [24]), it relates to underlying structural damage or myofibrillar dysfunction associated with the inclusions (or both). Given the subsarcolemmal location of the polysaccharide containing vacuoles in PSSM1, disruption of the dystrophin associated glycoprotein complex (DAGC), or its interaction with desmin and other sarcoplasmic intermediate filaments is plausible [17]. Alternatively the pathogenesis may relate to reduced energy substrate availability in the skeletal muscle of affected horses: although no differences in the activity of the cellular energy sensor, AMP-activated protein kinase, or in ATP or AMP concentrations, were found in affected horses [25], [26], increased inosine monophosphate concentration supports a role for disturbed energy metabolism [26], which could account for descriptions of myopathies in young foals with the disorder [21], [27].

As the clinical signs, and specifically the incidence of exertional rhabdomyolysis, vary between horses with PSSM1 [8], other factors must modify an individual animal's phenotype. For example, disease severity in PSSM1-affected horses increases in animals that also harbor a ryanodine receptor (RYR1) mutation [28]. Furthermore, the high prevalence of the R309H mutation within certain breeds means that homozygotes are commonly encountered; it is conceivable that some phenotypic variation may depend on allele copy number, with the disorder having incomplete dominance. Additional variation in phenotype may in part, result from differences in the degree to which breeds rely on oxidative and glycolytic muscle metabolism and specifically the different fibre type composition between breeds and animals. In one study of Quarter Horses with PSSM1, affected horses had 8% more type 2a fibres and 8% fewer type 2× fibres than controls [22]. This is in contrast to a subsequent study by the same group looking at Belgian horses with PSSM, where no difference in fibre type distribution between affected and control horses was found [29]. Furthermore, as dietary and exercise manipulation form the main stay of management recommendations for affected horses [30], it is also likely that environmental factors influence the disease phenotype.

Further investigation of skeletal muscle pathology in PSSM1 may help elucidate the mechanism by which the *GYS1* mutation results in the clinical signs, which in turn, may help direct novel treatments and disease management. The variable skeletal muscle phenotype in equine PSSM1 can be assessed by measurement of serum muscle enzyme activities and through skeletal muscle biopsy. In this manuscript we aimed to investigate the hypothesis that the severity of skeletal muscle pathology in PSSM1 would be greater in homozygotes than heterozygotes and controls. Furthermore, we hypothesised that horses with a higher proportion of polysaccharide-containing fibres, vacuolation or inclusions, would have higher resting, and/or post-exercise muscle enzyme activities. Specifically, we aimed to compare the proportions of fibres containing internalized nuclei, subsarcolemmal vacuoles and cytoplasmic polysaccharide inclusions in skeletal muscle between horses that were homozygous and heterozygous for the *GYS1* mutation, and control horses. Secondly, we aimed to compare fibre type composition between the different genotyped groups and to examine the integrity of dystrophin labeling adjacent to the subsarcolemmal vacuoles. Furthermore, given that some researchers have suggested a possible link between oxidant damage and exertional rhabdomyolysis in horses [31] we compared plasma vitamin E concentration between the groups.

## Materials and Methods

### All studies were approved by Auburn University's Institutional Animal Care and Use Committee (IACUC 2009-1536)

One hundred and twenty five Belgian and Percheron horses maintained with identical management (24 hour turn out) and diet, and owned by an Auburn University research facility, were genotyped (with owner permission). In brief, DNA was extracted from whole blood in EDTA using Nucleon's BACC kit^1^ according to the manufacturer's instructions. Genotyping was performed via a restriction fragment length polymorphism assay that identifies horses as homozygous (H/H), heterozygous (H/R) or wild type (R/R) for the *GYS1* R309H mutation [10]. Of the 125 horses, there were 9 homozygotes (H/H), 45 heterozygotes (H/R) and 71 unaffected animals (R/R). Three breed-, age- and sex-matched cohorts of 8 animals of each genotype (24 in total) were identified from these animals. All horses were not in current work, had 24-hour pasture turn out and were fed 2 kg per day of a supplementary concentrate diet and alfalfa as forage. The concentrate diet was designed to provide 22 MJ per day per horse, and contained 149 g/kg crude protein, 45 g/kg fat, 143 g/kg fibre and 165 g/kg starch.

Medical records, that included bi-annual routine biochemical analyses performed in January and July of each year from December 2003 to July 2009, were available for all 24 study horses. The number of available analyses depended on the time each animal had been within the herd from 5 to 14 samples (median of 9 samples). All resting serum creatine kinase (CK) and aspartate transferase (AST) activities were recorded and the median value calculated for each individual.

In order to measure resting muscle enzyme activity on the day of biopsy (see below), additional venous blood samples were collected by direct jugular venipuncture into heparinised and plain Vacutainers^2^ from the horses in each genotyped cohort. In addition, 4 horses (again matched for age and sex) from each genotype performed a standard exercise test consisting of 20 minutes of submaximal exercise (trot and canter work). Blood was collected immediately prior to, 4 and 24 hours post-exercise. All blood samples were immediately centrifuged at 4°C, and serum and heparinised plasma separated and stored at −80°C. Plasma vitamin E concentrations were analysed by a commercial laboratory (Veterinary Laboratories Agency, Shrewsbury, UK) by reverse phase HPLC and detected using a Shimadzu RF 551^3^ spectrofluorometric detector. Serum CK and AST activity were measured using a Roche Cobas C311 analyser^4^.

### Biopsy procedure

Four homozygotes, 8 heterozygotes and 6 control horses from the matched cohorts were available for open muscle biopsy. While standing under sedation, (combined detomidine and butorphanol) and mepivicaine subcutaneous local anaesthesia, a 3 cm×1 cm×1 cm sample of semimembranosus muscle was biopsied. Muscle was trimmed and immediately mounted onto a cork disc, with the fibres orientated vertically, and snap frozen in isopentane that had been pre-cooled in liquid nitrogen. These samples were transported on dry ice and subsequently stored at −80°C.

### Histopathology and immunohistochemistry

Fresh-frozen biopsy samples were sectioned at 8 and 16 µm, using a cryostat and stored at −80°C. Subsequently, sections were thawed, air dried for 20 minutes and then stained with haematoxylin and eosin (8 µm) and periodic acid Schiff (16 µm) with and without prior digestion with amylase [32]. A minimum of 200 fibres were examined in randomly-acquired images, captured with a ×20 objective, using an Olympus BX41 microscope with Micropublisher 3.3RTV Digital Camera (Qimaging). Haematoxylin and eosin-stained sections were evaluated to determine the percentage of fibres containing subsarcolemmal vacuoles, polysaccharide accumulations not associated with the sarcolemma, and internalised nuclei. The percentage of fibres containing diastase-resistant polysaccharide was determined in the periodic acid Schiff stained sections that had been pre-digested with diastase. The fibre type of affected fibres was identified by comparing serial sections stained, immunohistochemically for myosin heavy chains (below).

To characterise the fibre type muscle profiles, primary mouse anti-fast myosin heavy chain type 2a antibody (A4.74^5^; dilution 1∶5 in phosphate buffered saline (PBS)) or anti-slow myosin heavy chain antibody (MAB 1628^6^; dilution 1∶50 in PBS) were applied to 8 µm sections for 1 hour, to identify type 2a and type 1 muscle fibres respectively [33]. In addition, both antibodies were applied simultaneously to additional serial sections leaving type 2× fibres unstained. To characterise dystrophin expression and localisation, sections were incubated with mouse anti-DYS-2 antibody^7^ (dilution 1∶20 in PBS) for 1 hour. Biotinylated anti-mouse antibody^1^ (dilution 1∶ 200 in PBS) was applied secondarily followed by a tertiary peroxidase–streptavidin conjugate^8^ (dilution 1∶500 in PBS), both for 1 hour. A chromagenic substrate (Vectastain^9^) was then applied for 5 minutes followed by counterstaining with Gill's haematoxylin for 1 minute. Serial images stained for each fibre type were captured from randomly-selected areas using a ×10 objective and a minimum of 450 fibres were identified and typed.

### Statistical analysis

The normally distributed (Kolmogorov Smirnov test) age, breed and sex distribution data within groups were compared by one way analysis of variance (ANOVA) and chi square tests to confirm appropriate matching of groups. Subsequently, one-way ANOVA or Kruskal Wallis tests were used to compare data between genotyped groups following testing for normality. Where suitable, data that was not normally distributed was transformed prior to analysis. Post-hoc analysis included the use of Mann Whitney tests with Bonferroni correction. Associations between histopathological changes and muscle enzyme activities were assessed using Pearson's correlation coefficients or Spearman's correlation coefficients for normally, or non-normally distributed data respectively. All analyses were performed using statistical software^10^ and differences were considered statistically significant when P≤0.05.

## Results

### Signalment

There was no difference between the age, breed or sex of the three genotyped groups for either part of this study. Signalment data is presented in table 1. None of the horses included in this study had a history of muscle pain, cramping, sweating or exercise intolerance.

**Table 1:** Signalment of all horses participating in each section of the study, showing no significant differences between matched groups.

Part of study	Genotype	Age	P-value	Male	Female	P-value	Percheron	Belgian	P-Value
24 Horses	HH n = 8	9.6 (2.6)	0.99	5	3	0.47	3	5	0.11
	HR n = 8	9.8 (2.4)		3	5		1	7	
	RR n = 8	9.9 (2.7)		3	5		3	5	
Biopsy horses	HH n = 4	9.2 (3.4)	0.99	2	2	0.28	2	2	0.10
	HR n = 8	9.6 (2.4)		4	4		2	6	
	RR n = 6	9.7 (2.9)		3	3		2	4	
Exercise test	HH n = 4	9.3 (1.9)	0.98	2	2	0.56	2	2	0.56
	HR n = 4	9.5 (2.4)		1	3		1	3	
	RR n = 4	9.3 (1.9)		2	2		1	3	

### Histopathology and immunohistochemistry

**Figure 1:**
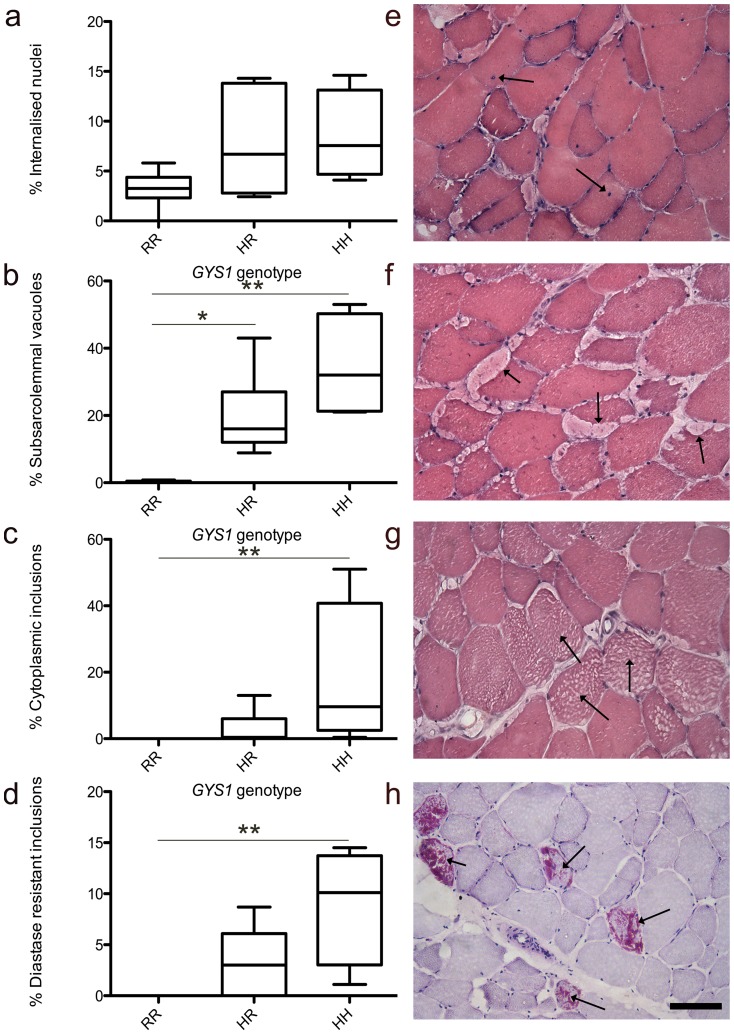
The effect of *GYS1* genotype on skeletal muscle histopathology. a–d: Boxplots illustrating the percentage of muscle fibres containing (a) internalised nuclei, (b) subsarcolemmal vacuoles, (c) cytoplasmic inclusions and (d) diastase resistant inclusions in horses homozygous (HH) and heterozygous (HR) for GYS1 mutation and controls (RR). * Denotes significant differences between individual groups (p<0.05), ** (p<0.01), following post- hoc analysis. e–h: Representative images of the pathology (arrows) identified (e) internalized nuclei, (f) subsarcolemmal vacuoles, (g) cytoplasmic inclusions and (h) diastase resistant inclusions (e–g: haematoxylin and eosin; h: Periodic acid Schiff following diastase digestion). Bar = 50 µm.

**Figure 2:**
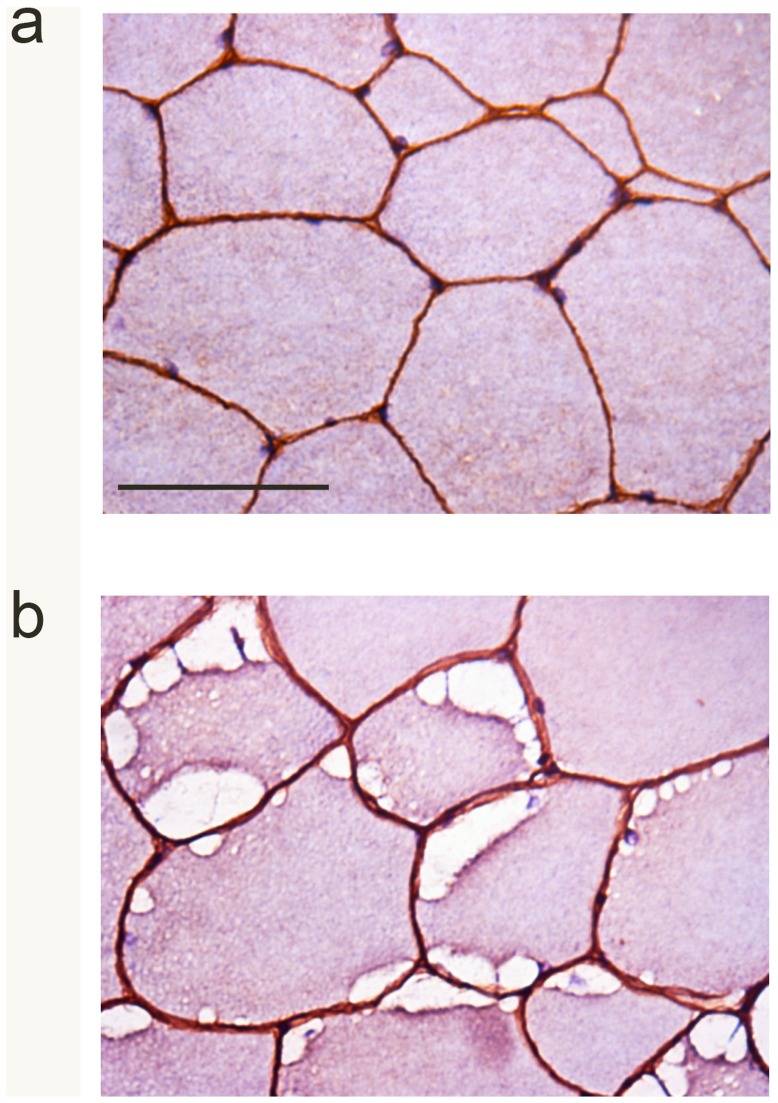
Dystrophin localisation. Representative images of dystrophin immunohistochemistry (brown staining) in skeletal muscle counterstained with haematoxylin and eosin from (A) a control horse and (B) a horse heterozygous for the *GYS1* mutation. Note the normal dystrophin expression and localisation in the region of the subsarcolemmal vacuoles (arrows). Bar = 50 µm.

A trend towards a higher percentage of muscle fibres containing internalised nuclei in the homozygous and heterozygous horses was observed when compared to the control horses and the difference between the 3 groups approached significance (p = 0.07) (figure 1a). A greater proportion of muscle fibres contained subsarcolemmal vacuoles (p<0.01) (figure 1b), cytoplasmic inclusions (p<0.01) (figure 1c) and diastase-resistant inclusions (p<0.01) (figure 1d) in the homozygous horses, when compared with the heterozygous and control groups. Following post-hoc analysis, significant differences between the homozygote and heterozygote groups were not identified for the percentage of fibres containing cytoplasmic inclusions (p = 0.15) or the percentage of fibres containing subsarcolemmal inclusions (p = 0.12), however the homozygotes had more fibres containing amylase-resistant inclusions (p = 0.05). There was a marked and significant correlation between the presence of subsarcolemmal vacuoles (rho = 0.86; p<0.001), and cytoplasmic inclusions (rho = 0.83; p<0.001), and the number of mutant alleles, and moderate, but significant correlation between the proportion of internalized nuclei and the number of mutant alleles (rho = 0.56; p = 0.02). Normal dystrophin localization and expression was identified at the sarcolemma in all muscle fibres, with no apparent reduction in expression adjacent to regions of subsarcolemmal vacuolation in either heterozygotes or homozygotes (figure 2).

### Fibre types

**Figure 3:**
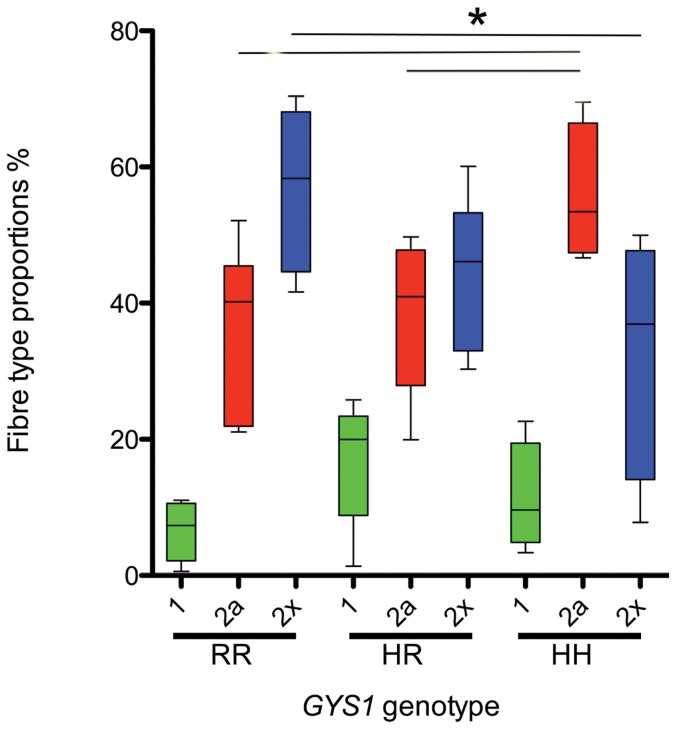
Muscle fibre type distributions. Boxplots illustrating the muscle fibre type composition of horses homozygous (HH) (n = 4), heterozygous (HR) (n = 8) and control horses (RR) (n = 6). * Denotes significant differences between individual groups (p<0.05) following post- hoc analysis.

There was no difference in the proportion of type 1 fibres within skeletal muscle (p = 0.06), between the 3 groups, however there was a significant difference between the proportion of type 2a (p = 0.04) and type 2× fibres (p = 0.03) within the muscle samples. Homozygotes had significantly more type 2a fibres than heterozygotes (p = 0.02) and con

trols (p = 0.03) and significantly fewer type 2× fibres than controls (p<0.05) (figure 3). There was no difference in the proportion of type 1 (p = 0.67) (HH median = 4.5%; (interquartile range (IR): 1.9–40.2); HR = 1.93% (0.4–4.91) RR = 0.01% (0–0.01)) or type 2× fibres (p = 0.20) (HH median = 10.6%; (interquartile range (IR): 4.9–17.3); HR = 15.2% (4.8–18.3) RR = 0.00% (0–0.4)) containing polysaccharide inclusions, however muscle from homozygous horses contained a significantly greater percentage of diastase-resistant polysaccharide-containing type 2a fibres (p = <0.0001) (HH median = 29.5%; (interquartile range (IR): 20.3–41.7); HR = 9.4% (4.5–15.0) RR = 0.00% (0–0.07)) than the other genotyped groups.

### Muscle enzyme activities

**Figure 4:**
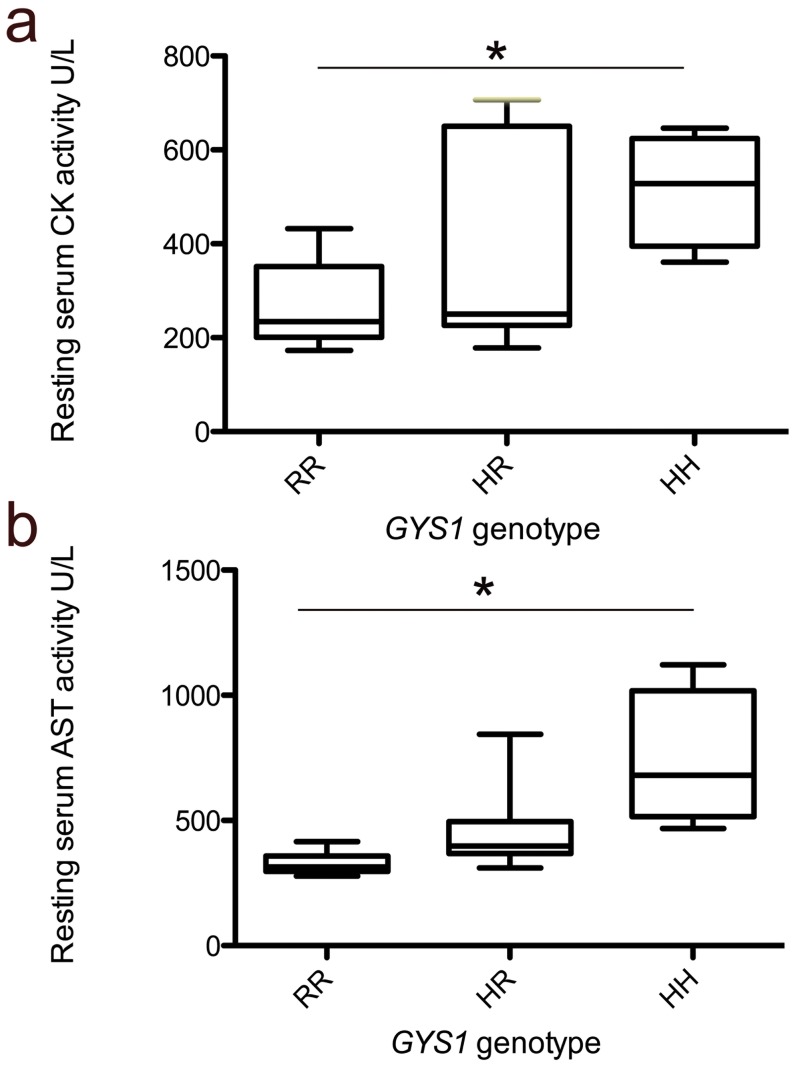
Resting muscle enzyme activities. Box plots illustrating the resting (a) CK activity and (b) AST activity for each GYS1 genotype (n = 8) (HH = homozygotes, HR = heterozygote, RR = control) * Denotes significant differences between individual groups (p<0.05) following post- hoc analysis.

**Figure 5:**
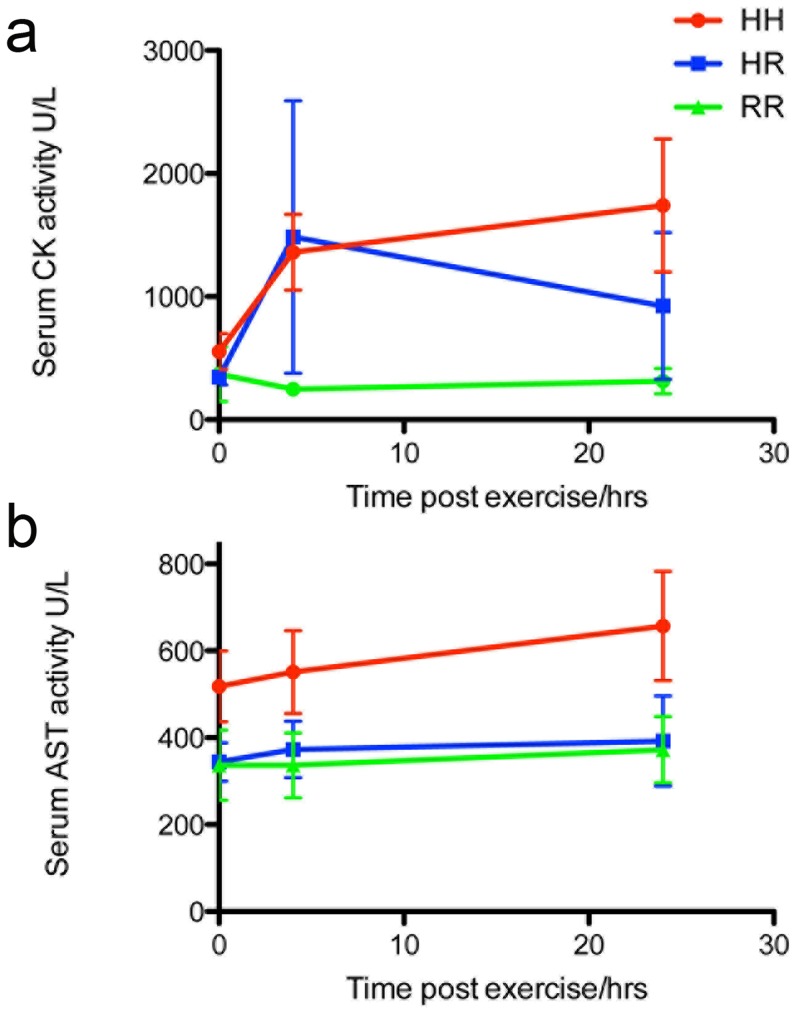
Pre- and post exercise muscle enzyme activites. Graph illustrating the pre and post exercise (a) CK activity and (b) AST activity for each GYS1 genotype (n = 4) (HH = homozygotes, HR = heterozygote, RR = control).

There was a significant difference between the groups' resting serum muscle enzyme activities (CK; p = 0.04 and AST; p = 0.01), with higher activities observed in homozygotes, followed by heterozygotes, in comparison with the control horses (Figure 4). There was a significant difference between CK activities at 4 hours post-exercise (p = 0.04) between the groups, with homozygotes having significantly higher activities (p<0.05) than control horses after *post hoc* analysis: this difference between genotypes also approached significance at 24 hours post exercise (p = 0.06) (Figure 5). There was no significant difference between heterozygous and control horses; indeed, there was considerable overlap between the two groups. When all horses with PSSM1 were combined (heterozygotes and homozygotes) and compared to control horses, a significant difference was detected in CK activity at 4 hours (p = 0.02) (PSSM1 median = 965 U/L (interquartile range (IR) 428–1989); RR = 253 U/L (IR 180–311)) but not 24 hours post-exercise (p = 0.07) (PSSM1 median = 1039 U/L (IR 360–2424); RR = 250 U/L (IR 161–528)). No difference in post-exercise AST activity was detected between the three groups at 4 hours post-exercise (p = 0.20) or 24 hours after exercise (p = 0.17) (figure 5), neither were there significant differences in AST activity after exercise in a combined PSSM1 heterozygote and homozygote group compared with controls (4 hours post-exercise; p = 0.22 (PSSM1 median = 422 U/L (IR 295–650); RR = 280 U/L (IR 235–494)); 24 hours post exercise; p = 0.46 (PSSM1 median = 478 U/L (IR 280–713); RR = 325 U/L (IR 263–528))). For all horses, there was a significant correlation between pre-exercise CK activity and that at 4 (rho = 0.68; p = 0.01) and 24 hours (rho = 0.77; p<0.01) post-exercise, and between pre-exercise AST activity and that at 4 (r^2^ = 0.97; p<0.001) and 24 (r^2^ = 0.52; p<0.01) hours post-exercise.

**Figure 6:**
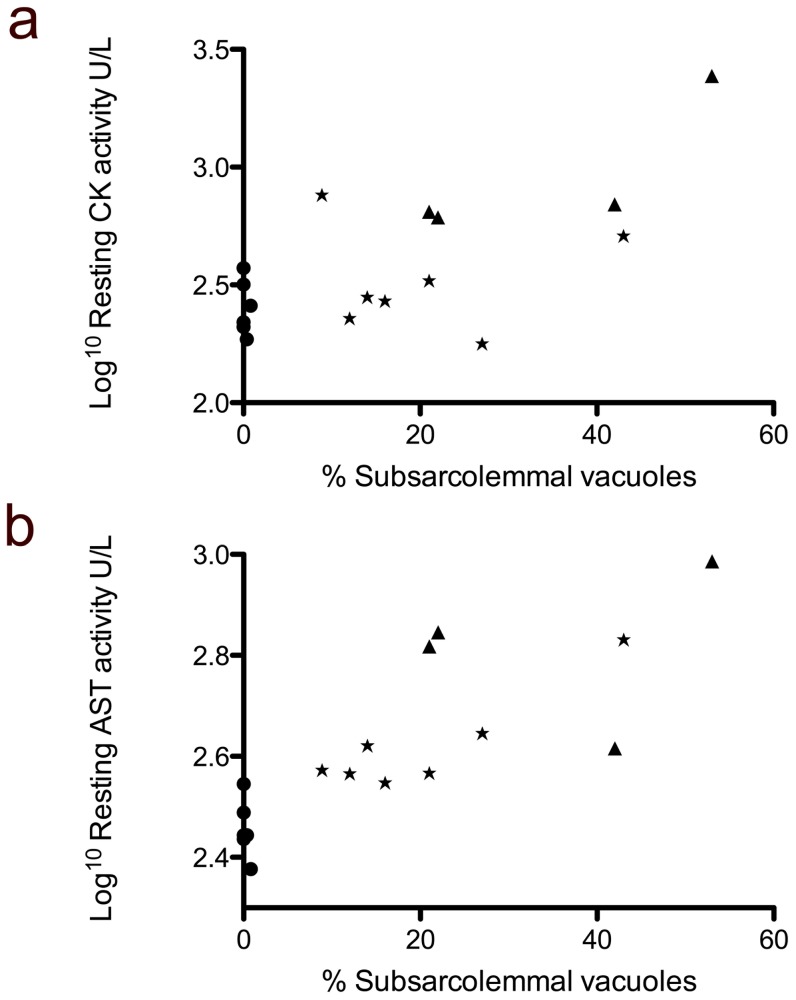
The correlation between muscle enzyme activity and muscle histopathology. Scattergraphs illustrating the correlation between the Log^10^ resting CK activity (A) and Log^10^ AST activity (B) and the percentage of fibres containing subsarcolemmal vacuoles. (HH = triangles, HR = stars, RR = circles) (n = 18).

There was a strong and significant association between the resting serum AST activity and the percentage of fibres with sarcoplasmic vacuoles (rho = 0.82; p = 0.01) and cytoplasmic inclusions (rho = 0.77; p = 0.01), but not internalised nuclei (rho = 0.44; p = 0.08) (figure 6), and a moderate but significant association between resting CK activity and subsarcolemmal vacuoles (rho = 0.51; p = 0.03), cytoplasmic inclusions (rho = 0.48; p = 0.05) and internalised nuclei (rho = 0.50; p = 0.04).

### Vitamin E

There was no difference between the plasma vitamin E concentrations of horses that were homozygous or heterozygous for the *GYS1* mutation or the control horses (p = 0.80), though control horses had higher vitamin E concentrations (mean +/−SD 9.86 µmol/l+/−1.92 (normal >4)) than the heterozygotes (9.10 µmol/l+/−2.44), which in turn had higher concentrations than homozygotes (8.96 µmol/l+/−3.56).

## Discussion

Equine PSSM1 manifests with a variety of clinical signs but the reason for this phenotypic variation is not clearly understood. A better understanding of the factors that act to modify the clinical presentation may provide clues as to the pathophysiology of the disease and ultimately aid in selecting appropriate treatment or management strategies. With many related and poorly understood glycogen storage diseases in humans [1], some findings may have relevance to the related human field. We have shown that skeletal muscle pathology in PSSM1 is typically more severe in homozygotes than heterozygotes and that horses with a higher proportion of polysaccharide-containing fibres and fibres with sub-sarcolemmal vacuolation have higher resting muscle enzyme activities.

This is the first study to demonstrate that the severity of muscle histopathological changes in PSSM1 depends on the underlying genotype and therefore supports the importance of genotyping affected individuals. Subsarcolemmal vacuolation and fibres with cytoplasmic inclusions were more prevalent in homozygotes than heterozygotes and both homozygous and heterozygous horses had more fibres containing internalised nuclei than control horses. Homozygotes had more fibres containing amylase-resistant polysaccharide inclusions than heterozygotes and in particular, a higher proportion of the mixed glycolytic-oxidative type 2A fibres containing amylase-resistant inclusions than heterozygotes. This excessive accumulation of polysaccharide aggregates appears directly related to increased activity of the mutant enzyme [10], and formation of less highly branched molecule [17].

In equine PSSM1, similar to the many human skeletal muscle glycogenoses, it is not yet understood how the presence of excessive glycogen and polysaccharide inclusions relates to the underlying disease. We found a strong and significant association between serum muscle enzyme activities and the proportion of fibres with polysaccharide aggregates and subsarcolemmal vacuoles. Although we cannot exclude the possibility that an underlying and unknown metabolic defect results in both the elevation in muscle enzyme activity and also the pathology independently, it seems plausible that muscle enzyme activities are higher in animals with more severe pathology because of physical disruption of the myofibrillar apparatus. We demonstrated normal dystrophin expression and normal sarcolemmal localisation adjacent to subsarcolemmal vacuoles in horses at rest; however it is conceivable that loss of interaction between the dystrophin-associated complex and the myofibrillar apparatus leads to fibre damage that is exacerbated by exercise (as seen in Duchenne muscular dystrophy) [34]. Alternatively, presence of glycogen or polysaccharide in itself may underlie the phenotype, as shown in neurons with artificially-induced excessive glycogen storage [35]. Indeed, although young foals with PSSM1 have been described (supporting a role for a metabolic defect), they also had higher muscle glycogen contents, sarcolemmal vacuolation and/or amylase-resistant polysaccharide from an early age [21], [27]. Perhaps regular and consistent exercise, which appear to be protective in PSSM1-affected horses and foals [21], [30] help enhance glycogen mobilization with concomitantly a reduction in vacuolation or cytoplasmic aggregation, and therefore less propensity to result in exercise-associated structural damage. We recently speculated that the constant work performed by the contracting heart protects against a cardiomyopathy in PSSM1, perhaps for the same reasons [36]. A future prospective study comparing skeletal muscle pathology in exercised vs non-exercised horses would be insightful.

In this study we demonstrated an association between the *GYS1* genotype and post-exercise CK and AST activities. Although there was a significant difference between the mean post-exercise CK activity of each group, the ranges of muscle enzyme activity overlap considerably, in particular between normal horses and heterozygotes and in some affected animals, it remains within the normal laboratory reference range both at rest and following submaximal exercise. Although we evaluated relatively few horses, resting or post-exercise muscle enzyme activity measurements appear to have low sensitivity and specificity. Some previous reports suggested muscle enzyme activity to be a useful screening test for PSSM1 [26], [37], however more recent work has demonstrated that many horses that are heterozygous for the *GYS1* mutation do not reach the previously suggested cut-off levels of 2–3 times resting muscle enzyme activity following exercise [38], as was observed in our study. In contrast to the findings in our study, Schwarz and co-workers [38] observed a significant difference between resting and post exercise AST activities and post exercise CK between heterozygous and control horses, although their study did not evaluate homozygotes. Until these discrepancies are better understood the genotyping of horses is recommended, even in the absence of significant elevations in post-exercise muscle enzyme activity, particularly in breeds with a high prevalence of the mutant *GYS1* allele.

Given the association between muscle enzyme activity and underlying pathology, CK and AST activity measurement may instead be useful prognostically for affected horses: the stronger association between histopathology and resting AST rather than CK activity supports the association of the pathology and chronic muscle damage. It is surprising that none of the horses in this study had shown clinical signs of muscle disease given the severity of the histopathology observed. This may reflect the low level of work performed by the horses included in this study or their permanent turn-out, as regular low intensity exercise is protective [30]. We suggest that horses that are worked hard may be more prone to exercise-associated muscle damage and hence rhabdomyolysis.

In this study we identified significant differences in fibre type proportions between the groups: horses with PSSM1 had a higher percentage of type 2a and fewer type 2× fibres, than controls and the extent of this difference was greatest for the homozygotes. This change in fibre type may be a reflect a shift from glycolytic towards more oxidative metabolism or be a consequence of type 2× fibre loss in affected horses. These findings are similar to those of Annandale and coworkers [22] who found 8% more type 2a fibres and 8% fewer type 2× fibres in Quarter Horses with PSSM; however, they contrast a subsequent study by the same group looking at Belgian horses with PSSM, where no difference in fibre type distribution between affected and control horses was found [29]. A fibre type shift with an increased proportion of type 2a fibres is an adaptive response to endurance training in human and equine athletes [39], [40], [41] however the horses in this study were not under training and were managed identically. The fibre type shift in affected horses may be an adaptive response, away from glycolytic and towards oxidative metabolism; in addition, the protective effect of regular exercise in PSSM1-affected horses would further encourage this adaptation [39], [40], [41]. Despite this, polysaccharide inclusions were still encountered in the mixed glycolytic-oxidative (2a) fibres, particularly in homozygotes. Given the similarities between PSSM1 and several human myopathies, further work to evaluate fibre type shifts (either induced or disease-associated) in human patients with skeletal muscle glycogenoses may be indicated.

Another possible mechanism of disease in glycogen storage diseases is free radical damage secondary to normal electron transfer, muscle inflammation or ischaemia [42]. Vitamin E is the principal defense for reactive oxygen species in tissues and skeletal muscle contains lower concentrations of vitamin E than other body organs [43], and may be more vulnerable to free radical damage, particularly in response to the large increases in metabolic demand that occur at exercise [42]. As in a previous study [44] we did not detect a significant difference between the plasma vitamin E concentrations between the different genotyped groups, however occasional homozygotes had plasma vitamin E concentrations below the normal reference range suggesting the possibility of excessive consumption. The lack of significance in this study may reflect the relatively small sample sizes; the potential beneficial effect of dietary supplementation with vitamin E for individuals with skeletal muscle glycogenoses, including horses with PSSM1 warrants further investigation.

In this study we have demonstrated a correlation between number of copies of the mutant *GYS1* allele, the severity of skeletal muscle pathology and the resting muscle enzyme activity. PSSM1 therefore has incomplete dominance and our work highlights the importance of genotyping affected animals to identify those individuals most likely to be more severely affected. Our work supports the possibility that there is a direct link between myofibre structural damage and the severity of the underlying phenotype. Furthermore, it suggests that management and treatment strategies aimed at maintenance of myofibre integrity may be beneficial in affected horses. Given the many similarities between PSSM1 and several human skeletal muscle glycogenoses, further study of this equine disorder may provide a useful model for work investigating the pathophysiology of this group of poorly understood, but important human diseases [45], [46].

### Manufacturer's addresses

^1^GE Healthcare, Buckinghamshire, UK.

^2^Becton, Dickinson U.K. Limited, Oxford, UK.

^3^Shimadzu UK Ltd, Milton Keynes, UK.

^4^Roche Diagnostic Corporation, Indianopolis, USA.

^5^Developmental Studies Hybridoma Bank, University of Iowa, Iowa, USA.

^6^Millipore, Watford, UK.

^7^Novacastra reagents, Leica Microsystems (UK) Ltd, Buckinghamshire, UK.

^8^Stratech Scientific limited, Newmarket, Suffolk, UK.

^9^Vector Laboratories, Burlingame, California, USA

^10^SPSS Version 18, IBM, Middlesex, UK.
